# Novel AI driven approach to classify infant motor functions

**DOI:** 10.1038/s41598-021-89347-5

**Published:** 2021-05-10

**Authors:** Simon Reich, Dajie Zhang, Tomas Kulvicius, Sven Bölte, Karin Nielsen-Saines, Florian B. Pokorny, Robert Peharz, Luise Poustka, Florentin Wörgötter, Christa Einspieler, Peter B. Marschik

**Affiliations:** 1 University Medical Center Göttingen, Child and Adolescent Psychiatry and Psychotherapy, 37075 Göttingen, Germany; 2 Division of Phoniatrics, Research Unit interdisciplinary Developmental Neuroscience, Medical University of Graz, 8036 Graz, Austria; 3 Leibniz ScienceCampus Primate Cognition, 37075 Göttingen, Germany; 4 Georg-August University Göttingen, Third Institute of Physics-Biophysics, 37077 Göttingen, Germany; 5 Department of Women’s and Children’s Health, Karolinska Institutet, Center of Neurodevelopmental Disorders (KIND), 113 30 Stockholm, Sweden; 6 University of California, David Geffen School of Medicine, Los Angeles, CA 90095 USA; 7 University of Augsburg, EIHW-Chair of Embedded Intelligence for Health Care and Wellbeing, 86159 Augsburg, Germany; 8 Department of Mathematics and Computer Science, Eindhoven University of Technology, 5600 MB Eindhoven, The Netherlands

**Keywords:** Movement disorders, Predictive markers, Neurological disorders, Translational research

## Abstract

The past decade has evinced a boom of computer-based approaches to aid movement assessment in early infancy. Increasing interests have been dedicated to develop AI driven approaches to complement the classic Prechtl general movements assessment (GMA). This study proposes a novel machine learning algorithm to detect an age-specific movement pattern, the fidgety movements (FMs), in a prospectively collected sample of typically developing infants. Participants were recorded using a passive, single camera RGB video stream. The dataset of 2800 five-second snippets was annotated by two well-trained and experienced GMA assessors, with excellent inter- and intra-rater reliabilities. Using OpenPose, the infant full pose was recovered from the video stream in the form of a 25-points skeleton. This skeleton was used as input vector for a shallow multilayer neural network (SMNN). An ablation study was performed to justify the network’s architecture and hyperparameters. We show for the first time that the SMNN is sufficient to discriminate fidgety from non-fidgety movements in a sample of age-specific typical movements with a classification accuracy of 88%. The computer-based solutions will complement original GMA to consistently perform accurate and efficient screening and diagnosis that may become universally accessible in daily clinical practice in the future.

## Introduction

Research on early motor functions has a long history. After the monumental detachment from the reflex-focused approach, Heinz Prechtl pioneered a novel route some 30 years ago to systematically investigate *spontaneous* movements (i.e., free from external stimuli) in preterm and term infants^1,2^. The investigation indicated a qualitative deviation, but not a quantitative difference in the movement patterns pointing to neurological dysfunctions^3–6^. This was the starting point for the development of the Prechtl general movement assessment (GMA).

GMA became internationally known in 1997 with the first publication on this topic by Prechtl et al.^7^. It is a clinical reasoning approach based on visual gestalt perception of normal vs. abnormal infant movements in the entire body, hence the term general movements (GMs). Initially a promising new method for evaluating the integrity of the young nervous system via the assessment of an overt behavior, GMA has become one of the most widely-used and reliable tools for the detection of cerebral palsy during early infancy^8–11^.

**Figure 1:**
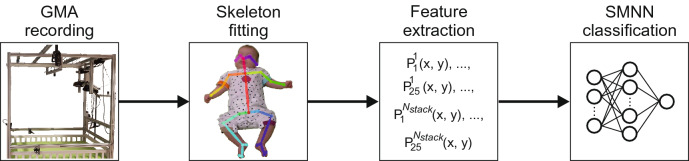
Overview of the algorithm’s process pipeline.

From the 9$\mathrm{th}$ week postmenstrual age to approximately 20 weeks of postterm age, fetuses/infants show a distinct repertoire of endogenously generated (i.e. independent of a sensory input) movement patterns such as startles, GMs, breathing movements, yawning, and sucking^12,13^. Normal GMs are variable sequences of neck, trunk, leg, and arm movements, with gradual beginnings and endings and of changing intensity, force, and speed^1^. Before term age, GMs are referred to as fetal or preterm GMs, whereas movements observed between term age and approximately 6–8 weeks of postterm age are termed writhing movements (WMs). Normal WMs can last between seconds and several minutes. They predict a normal neurodevelopmental outcome with a negative likelihood ratio (LR) of 0.04^14^. During the WMs period, the abnormal GM patterns include *poor repertoire* (PR), with LR+ = 0.61; *cramped-synchronized* (CR), with LR+ = 45; or the very rare *chaotic* patterns^14^.

WMs gradually disappear during the second month postterm, and a new pattern of GMs, the Fidgety Movements (FMs), emerges. Normal FMs are small amplitude movements of moderate speed with variable acceleration of the neck, trunk and limbs in all directions. They are continually observable during active wakefulness, yet are disrupted during episodes of fussing and crying. Normal FMs are predictive of normal neurodevelopment (LR− = 0.05)^14^. With a sensitivity of 98% and specificity of 91%^11^, the *absence* of FMs at 3–5 months of postterm age is the best predictor of later development of neurological impairments (e.g., cerebral palsy; LR+ > 51^14^), exceeding the predictive power of cranial ultrasound and other neurological examinations, and is comparable to brain magnetic resonance imaging^8,11^. FMs gradually fade out around 20 weeks of postterm age when voluntary movement patterns become predominant^15,16^.

General movements are generated by the central pattern generators (CPGs), a neural network, which is most likely located in the brainstem. Variability in the motor output is achieved by supraspinal projection, inhibition, and, most importantly, modulation of CPG activity^17,18^. If the CPGs exhibit reduced modulation, less variable, i.e. abnormal, movements are shown, indicating fetal or neonatal compromise^15,19^.

Standard GMA requires observation of merely 2–5 min of an infant’s spontaneous movements by trained assessors^15^. While brain-imaging and EEG decipher neurological structure and functions at the analytical level, GMA evaluates the functional brain as a whole. Compared to other tools (e.g., MRI/DTI, EEG, fNIRS), GMA is non-intrusive and easy to apply, while being highly informative and valid. As an efficient and reliable diagnostic tool, GMA is particularly suitable for low resource settings. In addition to its application in infants with perinatal brain injury, GMA has been widely applied to assess young infants with various neurodevelopmental and genetic disorders, as well as congenital infections^20–24^.

Although gestalt perception is a powerful tool for analyzing phenomena with complex and changeable, albeit expected characteristics, it is contingent on the observer’s skills and experiences. Like all man-powered assessments, GMA is vulnerable to human factors (e.g., fatigue and other physical influences, limited skills or experience, biases and subjectivity) and environmental influences. Although the reliability of GMA has repeatedly proved high for well-trained assessors, with inter-rater agreement ranging between 89 and 98%^14,25–27^, this degree of excellence does require specific high-quality training, with continuous practice and re-calibration of the assessors. Despite that GMA is urgently needed as a highly efficient and valid tool for the young population as well as for the society, the cumulative cost and effort required for maintaining adequate standard practices among GMA assessors can add up and become quite challenging. As such, GMA has yet not been scaled up widely enough as ought to be (e.g., in worldwide routine medical procedures and well-child care). As automated machine learning (ML) approaches can avoid the influence of unfavorable human and environmental factors, they might have the potential to augment the merits of GMA and boost its application.

As a consequence, the last decade has evinced a boom of computer-based approaches to complement classic GMA^28–30^. Leveraging ML approaches to track infants’ movements, researchers have applied different types of sensors either by attaching them directly on the infant’s skin or by placing them into the wearables. For example, in 2008, an electromagnetic tracker system was introduced for cerebral palsy detection, where a marker was placed on each of the four extremities and their positions in space were measured^31,32^. A sensitivity of 90% and a specificity of 96% had been reported^33^.

In recent years, more promising, wireless measurement devices were presented to the scientific community. For example, a so-called “chest unit” has been invented to be placed directly on the skin^34^. It consists of a 3 degrees of freedom (DoF) accelerometer, a thermometer, an ECG system, and a pulse oximetry module. Similarly, a “smart jumpsuit” featuring 4 IMU sensors with 6 DoF was presented^35^. Based on these sensors, basic posture (accuracy of 95.97%) and movement recognition (accuracy of 76.73%) was performed. In another work, 2 IMUs with 6 DoF were placed on the infant’s feet^36^. These two sensors were reported to be able to differentiate typical from atypical movements^36^.

**Table 1:** Detailed information of the $N=45$ participants.

	Mean	Standard deviation	Min	Max	Percentiles		
					25	50	75
Gestational age (weeks)	39.0	1.3	35	41	38	39	40
Birth weight (g)	3440.9	382.1	2500	4416	3300	3448	3680
Birth length (cm)	51.5	2.1	46	56	50	51	53
**APGAR score**							
1 min	8.9	0.8	4	10			
5 min	9.9	0.6	6	10			
10 min	10.0	0.2	9	10			

For participant with ID 28 no APGAR scores could be obtained. The APGAR score^37^ was developed to evaluate a newborn’s health condition and the potential need of neonatal care based on five categories (Appearance, Pulse, Grimace, Activity, Respiration). A score $\ge 7$ is considered normal, scores ranging between 4 and 6 are classified as fairly low, and scores $\le 3$ as critically low^37,38^. The APGAR assessment is routinely applied three times, i.e. 1, 5, and 10 min after birth.

Although these implementations are able to report accurate localizations of the (x, y, z)-position of the IMUs, the sensors and the wearables might interfere with the infant’s spontaneous movements^39^. Moreover, full body tracking is impossible with such methods. In recent years, advancements in camera technology, as well as in computer vision have enabled body part tracking via 2D RGB cameras. This fully non-intrusive approach (i.e. no marker on the infant’s body) allows tracking the infant’s free and spontaneous movements as required by GMA. More importantly, not only the position of the single points, i.e. the IMUs, but also the position of all joints of the infant can be captured.

These non-intrusive methods can be divided into two approaches. First, only certain body parts or features are tracked and classification is based on their motion patterns. Second, a full pose of the infant in form of a skeleton model is recovered and classification is based on the skeleton’s movement characteristics.

For the first approach, numerous algorithms exist and show satisfying results. For example, by counting pixels of moving body parts and computing their mean and standard deviation, cerebral palsy was reported to be detected with a sensitivity of 85% and a specificity of 71%^40^. A more refined approach of the same technique used logistic regression for automated classification of fidgety movements^41^. In a more advanced method, the infant’s body was segmented into pixel clusters, which were tracked, and an accuracy of 87% was achieved^42^. Similarly, one can track the legs and feet of infants and use these features for classification. For different movement types, a precision ranging 85–96% and recall ranging 88–94% were obtained^43^. In 2020, deep learning methods have been introduced into the automated GMA field and showed a classification accuracy of 84.52% for fidgety movements on low birth weight participants^39^.

For the second approach, targeting pose estimation, two methods currently exist as the de-facto standard. First, DeepLabCut allows for markerless tracking of predefined body points^44^. It contains a pre-trained neural network where the user manually defines and labels tracking points on sample images, which are then used for transfer learning. As a result, DeepLabCut can track unknown points in previously unseen data, for example data from animals. Similarly, OpenPose is also a deep learning-based approach. Different from DeebLabCut, which uses human annotations for tracking, OpenPose was being trained on a human skeleton model^45^. For a given RGB image, the network outputs (x, y)-positions of skeleton points. In our current work, OpenPose is used since it does not require manual labeling of body parts that need to be tracked. An example image can be seen in Fig. 3. Both, DeepLabCut and OpenPose, work on 2D RGB image streams from single or multiple cameras. Current state-of-the-art methods use RGB cameras for full pose recovery^28^. Other approaches utilize RGB-D depth sensors^28,46,47^.

In this paper, we present a method for automated recognition of fidgety movements with a new feature vector. We utilize OpenPose^45^ for full body tracking from single 2D RGB images, from which a feature vector is constructed. No multi-camera setup, depth perception sensor, or motion capture system is required. As a new feature vector for classification, a normalized skeleton is used, i.e. raw (*x*,  *y*)-positions of 25 extracted skeleton points (see Fig. 3). These features can be easily interpreted by humans. Classification is performed using a shallow multilayer neural network (SMNN). The choice of a shallow network architecture was determined by the fact that, in general, shallow network architectures perform well for relatively small input vectors and for relatively small amounts of training data. Usually, deep neural network architectures which directly work on images, which makes the input space huge (e.g. image of 200 $\times$ 200 corresponds to 40,000 inputs), require a lot of training samples (in the order of 100,000 and more). In this work, the dataset is rather small, but, as we do not use raw images as input, the feature vector is also quite small compared to images, i.e., it consists of coordinates (x, y) of 25 skeleton points times number of frames (around 50). Another advantage of shallow network architectures are the fast training and inference times. In Fig. 5 it is shown, that our networks can be trained within less than 10 min and perform inference in 20 ms, whereas training very deep learning architectures usually takes days or weeks.

Importantly, while previous ML approaches mainly focused on differentiating typical from atypical GMs, here we present a new perspective of research aiming at detecting distinguished age-specific typical movement patterns. In particular, we aim at an automated detection and classification of *presence* vs. *absence* of FMs in typically developing young infants.

This paper is structured as follows. In the next section, we first introduce the dataset and the participants, followed by the presentation of our novel framework. Afterwards, our results are presented and discussed from both the technological and clinical perspectives.

## Approach

Data acquisition was conducted at iDN’s BRAIN*tegrity* lab at the Medical University of Graz (Austria). Data analyses were performed at the Systemic Ethology and Development Research Unit, Department of Child and Adolescent Psychiatry and Psychotherapy at the University Medical Center Göttingen, Germany. The algorithm’s pipeline is shown in Fig. 1, and consists of four steps: Data recording using a single RGB camera, full body tracking using OpenPose^45^, feature extraction, and classification using an SMNN. Details on the movement recordings are presented in the next section. Afterwards, the full body tracking using a skeleton model is explained. The skeleton points are then used as features (inputs) for the neural network to perform classification, which is explained in the last subsection of the “Approach” section.

**Table 2:** Description of dataset splitting.

	Number of snippets			Participant IDs
	FM+	FM−	Total	
Total	956 (53.6%)	828 (46.4%)	1784	
Validation	237	202	439 (24.6%)	7, 10, 14, 15, 17, 18, 34, 44, 47, 50, 51
**Set 1**				
Training	471	414	885 (49.6%)	1, 5, 8, 9, 12, 16, 19, 20, 21, 22, 23, 27, 28, 29, 30, 33, 36, 37, 38, 39, 40, 42, 43, 45, 48, 49
Testing	248	212	460 (25.8%)	3, 4, 26, 31, 32, 35, 41, 46
**Set 2**				
Training	482	412	905 (50.7%)	1, 4, 8, 16, 19, 21, 23, 26, 28, 29, 31, 32, 33, 36, 39, 42, 45, 46, 49
Testing	237	203	440 (24.7%)	3, 5, 9, 12, 20, 22, 27, 30, 35, 37, 38, 40, 41, 43, 48
**Set 3**				
Training	487	425	912 (51.1%)	1, 3, 4, 5, 9, 16, 19, 20, 23, 26, 27, 29, 31, 32, 38, 39, 40, 42, 49
Testing	232	201	433 (24.3%)	8, 12, 21, 22, 28, 30, 33, 35, 36, 37, 41, 43, 45, 46, 48
**Set 4**				
Training	483	422	905 (50.7%)	1, 3, 4, 8, 9, 16, 19, 20, 23, 26, 28, 31, 32, 35, 37, 38, 40, 41, 43, 46
Testing	236	204	440 (24.7%)	5, 12, 21, 22, 27, 29, 30, 33, 36, 39, 42, 45, 48, 49
**Set 5**				
Training	486	421	907 (50.8%)	1, 4, 5, 9, 19, 20, 21, 23, 26, 27, 29, 31, 35, 36, 38, 40, 41, 42, 45, 46, 48
Testing	233	205	438 (24.6%)	3, 8, 12, 16, 22, 28, 30, 32, 33, 37, 39, 43, 49

The validation set was used for hyperparameter tuning whereas sets 1–5 were used for cross-validation and evaluation of network architectures.

### Participants

From 2015 to 2017, 51 newborns (26 females, 25 males) from Graz and its surroundings were recruited for our prospective longitudinal study “Early Human Development: Pilot study on the 3-Month-Transformation”^48^ on neuromotor, visual, and verbal development. We included infants according to the following criteria: uneventful pregnancy, uneventful delivery at term age (> 37 weeks gestation), singleton birth, appropriate birth weight, uneventful neonatal period, inconspicuous hearing and visual development. Besides, no mother of the infants had either current or history of alcohol or substance abuse (see Table 1 for participants’ information). Infants were brought to our lab biweekly from 4 to 16 weeks postterm. Postterm ages for the seven consecutive sessions were: T1 $28 \pm 2\ \mathrm{days}$, T2 $42 \pm 2\ \mathrm{days}$, T3 $56 \pm 2\ \mathrm{days}$, T4 $70 \pm 2 \ \mathrm{days}$, T5 $84 \pm 2\ \mathrm{days}$, T6 $98 \pm 2 \ \mathrm{days}$, and T7 $112 \pm 2\ \mathrm{days}$.

One infant was excluded from the current analysis due to a diagnosed medical condition at age 3 years. Another five infants were excluded due to incompleteness of recordings within the required age intervals (please see below). The final sample size was thus 45. None of the 45 participants was reported to have any developmental impairment by the time of data analysis.

The study was approved by the Institutional Review Board of the Medical University of Graz, Austria (27-476ex14/15) and all experiments were performed in accordance with the approved guidelines and regulations. Parents were informed about all experimental procedures and the purpose of the study and gave their written informed consent for participation and publication of results.

### Materials and dataset

The assessment of the developmental trajectory of GMs, from writhing to fidgety movements^15^, was part of our study protocol with the afore-mentioned seven consecutive repeated-measure sessions^48^. Procedures of standard recording of GMs were reported elsewhere^48^. For this study, we used data from T1 as “pre-fidgety period” and T5-7 as “fidgety period”^15,16^.

All accessible videos (i.e., infants were awake and active, without pacifier, overall not fussy or crying) during recording of T1 (N = 838) and T5-7 (N = 946) are included. For training of the SMNN, each video was first cut into brief chunks. During the piloting period, we determined the shortest length of each video snippet to be 5 s, a reasonable duration of unit for machine learning, as well as a minimum length of video for human assessors feeling confident to judge whether the fidgety movement is present (FM+) or absent (FM−) on each snippet, providing a dichotomous classifier for the machine learning process.

**Figure 2:**
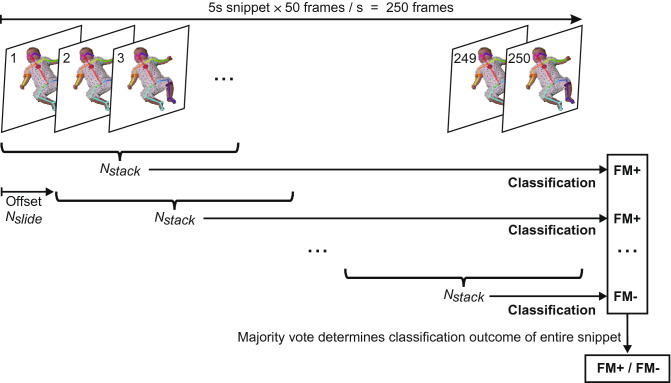
An overview of the feature extraction and classification procedure. One snippet has a length of $250\ \mathrm{frames}$. $N_{stack} = 52 \ \mathrm{frames}$ are concatenated to one input vector—i.e. the (*x*,  *y*)-values of $52 \ \mathrm{frames}$ frames are stacked together to form one input vector. The offset between two input vectors is $N_{slide} = 12\ \mathrm{frames}$, resulting in a sliding window approach. Each input vector is classified independently. The final decision is made based on uniform majority vote.

Out of the total available 19,451 snippets, 2800 (1400 from T1, the pre-fidgety period, and the rest from the T5-7, the fidgety period) were randomly selected for annotation by human assessors. Two experienced GMA assessors (DZ and PBM), blind of the ages of the infants, evaluated all the randomly ordered 2800 five-second snippets separately, labeling each snippet as “FM+”, “FM−”, or “not assessable” (i.e., the infant during the specific 5 s was: fussy/crying, drowsy, yawning, refluxing, over-excited, self-soothing, or distracted by the environment, all of which distort infants’ movement pattern and shall not be assessed for GMA^15^). The inter-rater agreement of the two assessors was excellent (Cohen’s kappa $\kappa = 0.97$, for classes FM+ and FM−). The intra-assessor reliability by re-rating 280 randomly-chosen snippets (i.e. 10% of the sample) was Cohen’s kappa $\kappa = 0.85$ for assessor 1, and $\kappa = 0.95$ for assessor 2 for the classes FM+ and FM−. Snippets with discrepant labeling by the assessors were excluded ($N = 316$). The snippets labeled as “not assessable” ($N = 700$; 417 from the pre-fidgety period, and 283 the fidgety period) by either assessor were also excluded from further analysis. A remaining total of 1784 snippets were labeled identically by both assessors: either FM+ ($N = 956$, of which 19 came from T1, the pre-fidgety period), or FM− ($N = 828$, of which 819 came from T1).

These 1784 snippets were used for the machine learning procedure. Using a genetic algorithm implementation^49^ of the knapsack problem^50^, the snippets were separated into validation (about 25%), training (about 50%), and testing sets (about 25%), so that snippets of each participant appear in only one of the three sets. This way we generated one validation set for feature and learning parameter tuning, whereas training and testing sets were generated fives times to perform cross-validation for evaluation of different network architectures. An overview of the datasets is presented in Table 2. For the current study, we identify participants by their IDs (1–51). As mentioned above, six of the participants (ID 2, 6, 11, 13, 24, 25) were excluded.

### Body tracking and feature extraction

**Figure 3:**
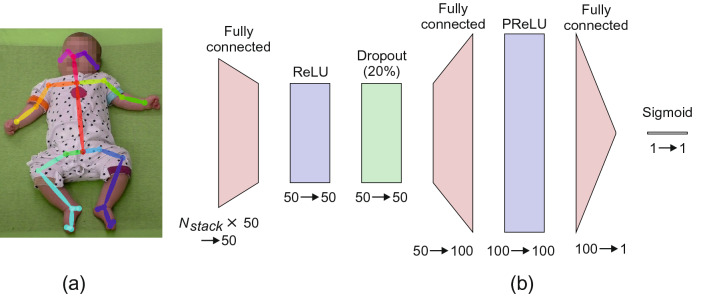
(**a**) An example frame with 25-point skeleton overlay and (**b**) a schematic diagram of the SMNN 5 network architecture.

For body tracking, the OpenPose algorithm was used^45^. OpenPose is a deep learning method, which extracts a 25-point skeleton from image frames. Each skeleton point consists of a 2 dimensional position (*x*, *y*), leading to a 50-point vector per frame. To ensure that the learning algorithm does not take the infant’s size into account, the skeleton is scaled to 1. If joints are not correctly identified by OpenPose, usually because of occlusions, values are filled with 0. One skeleton sample is shown in Fig. 3.

An overview of the feature extraction process is displayed in Fig. 2. One video snippet has a length of 5 s with a sampling rate of 50 fps, resulting in a total of 250 frames. One input vector for the SMNN is constructed of multiple, stacked frames. The number of stacked frames $N_{stack}$ is a hyperparameter of the feature vector and was optimized on the validation set. For example, $N_{stack} = 52$ will result in an input vector sized $50 \times 52 = 2600$ values. This vector corresponds to 1.04 s of the video snippet. The next input vector is generated using a sliding window approach. The offset between the vectors is a second hyperparameter, $N_{slide}$, which was also optimized on the validation set.

### SMNN learning and classification

In this study, we compared nine different SMNN architectures as shown in Table 3 where we varied the number of hidden layers (from one to three) and the number of neurons per layer (50, 100 and 200). SMNN 1–3 consists of one hidden layer, SMNN 4–6 of two hidden layers, and 7–9 of three hidden layers. In the first hidden layer rectified linear units (ReLU)^51^ were used, whereas in the second and third hidden layers parametric ReLU units (PReLU) were used. For regularization and preventing the co-adaptation of neurons, a dropout layer (20%) was used between hidden layers in SMNN 4–9, too^52^. Finally, in the output layer we used a neuron with a sigmoid transfer function. A visualisation of SMNN 5 architecture is presented in Fig. 3. The Adam optimizer^53^ was used with the learning rate $\alpha = 0.001$ and the time scale parameters $\beta _1 = 0.9$ and $\beta _2 = 0.999$.

**Table 3:** Description of SMNN architectures.

SMNN	Hidden layer	Neuron type	Drop out	Hidden layer	Neuron type	Drop out	Hidden layer	Neuron type	Output layer	Neuron type
1	$N_{stack} \times 50 \rightarrow 50$	ReLU							1	Sigmoid
2	$N_{stack} \times 50 \rightarrow 100$	ReLU							1	Sigmoid
3	$N_{stack} \times 50 \rightarrow 200$	ReLU							1	Sigmoid
4	$N_{stack} \times 50 \rightarrow 50$	ReLU	20%	50	PReLU				1	Sigmoid
5	$N_{stack} \times 50 \rightarrow 50$	ReLU	20%	100	PReLU				1	Sigmoid
6	$N_{stack} \times 50 \rightarrow 50$	ReLU	20%	200	PReLU				1	Sigmoid
7	$N_{stack} \times 50 \rightarrow 50$	ReLU	20%	50	PReLU	20%	50	PReLU	1	Sigmoid
8	$N_{stack} \times 50 \rightarrow 50$	ReLU	20%	100	PReLU	20%	100	PReLU	1	Sigmoid
9	$N_{stack} \times 50 \rightarrow 50$	ReLU	20%	200	PReLU	20%	200	PReLU	1	Sigmoid

Numbers correspond to the number of neurons in each layer. For example, SMNN 1 consists of one hidden linear layer with 50 ReLU neurons and a linear output layer with one sigmoid neuron. $N_{stack} \times 50$ denotes dimension of the input to the first hidden layer.

There are several hyperparameters related to the discussed network architectures. Two hyperparameters are with respect to the feature vector, i.e., number of frames per input vector $N_{stack}$ and the offset between two consecutive input vectors $N_{slide}$ (see Fig. 2), and another parameter is related to the training procedure of the SMNN, i.e., batch size $N_{batch}$. These hyperparameters were tuned as follows. First, a set of initial values was determined heuristically. Second, one of the parameters, e.g., batch size $N_{batch}$ was iterated over some range (see Fig. 6) while the other paraemters were kept constant. Based on the True Positives (TP), True Negatives (TN), False Positives (FP), and False Negatives (FN) the True Positive Rate (TPR) was computed as $\mathrm {TPR} = \mathrm {TP} / (\mathrm {TP} + \mathrm {FN})$ and similarly the False Positive Rate as $\mathrm {FPR} = \mathrm {FP} / (\mathrm {TN} + \mathrm {FP})$. Best values are $\mathrm {FPR} = 0$ and $\mathrm {TPR} = 1$, where $N_{batch}$ was chosen with the minimum distance $d = \sqrt{\left( 1-\mathrm {TPR}\right) ^2 + \left( 0-\mathrm {FPR}\right) ^2}$. Afterwards, $N_{batch}$ was kept constant, and the procedure was repeated for $N_{stack}$, and $N_{slide}$. For this ablation study SMNN 5 with the training set 1 and the validation set was used (see Table 2). The parameters were kept constant for all other experiments.

## Results

In this section, we present the results for the performance evaluation of the proposed approach and discussed network architectures. We first justify the selection of the hyperparameters. Next, we present the classification performance of the proposed networks.

Results of an ablation study for the hyperparameter tuning are shown in Fig. 6 where we show performance scores after convergence of the learning for each parameter. The best performance with respect to TPR and FPR, and classification accuracy was obtained with $N_{batch} = 3968$, $N_{stack}= 52$, and $N_{slide} = 12$.

**Figure 4:**
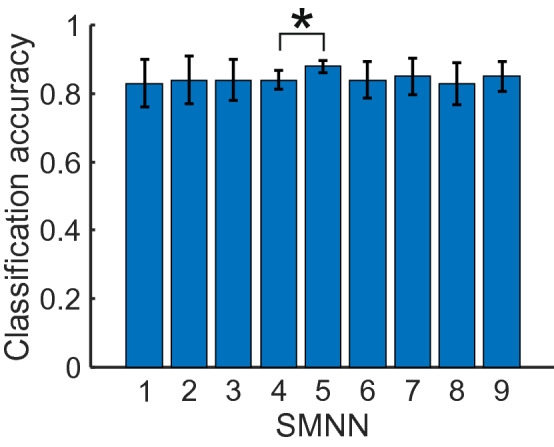
Comparison of SMNN architectures (Table 3) on classification accuracy. Mean classification accuracy obtained on five cross-validation test sets (Table 2) are shown for each model. Error bars denote confidence intervals of mean (95%). Mean difference of SMMN 5 and SMMN 4 is statistically significant ($t-test$, $p=0.0381$). Differences of all other means are not statistically significant ($t-test$, $p>0.05$ for all other pairs).

The results and corresponding performance scores of all nine SMNNs averaged over the five cross-validation test sets are shown in Table 4 and Fig. 4. Network SMNN 1–3 contains one, SMNN 4–6 contains two, and SMNN 7–9 contains three hidden layers, respectively. As shown in Fig. 4 all network architectures lead to similar classification performance with one exception where SMNN 4 performs worse than SMNN 5 ($t-test$, $p=0.0381$). Difference off all other means are statistically not significant ($t-test$, $p>0.05$ for all other pairs). However, the SMNN 5 network has much smaller variance (see also Table 4) across five cross-validation test sets as compared to the other SMMN networks making it most stable with respect to classification performance on new datasets (i.e., datasets not used in training).

**Table 4:** Comparison of SMNN 1–9 architectures (see Table 3).

SMNN	TP	FP	FN	TN	Sensitivity		Specificity		Precision		Acc.	F1-Score	
					FM+	FM−	FM+	FM−	FM+	FM−		FM+	FM−
1	209.00	46.60	28.20	158.40	0.88	0.77	0.77	0.88	0.83	0.86	0.83	0.85	0.80
	(14.80)	(39.48)	(18.95)	(41.75)	(0.08)	(0.20)	(0.20)	(0.08)	(0.10)	(0.06)	(0.08)	(0.06)	(0.13)
2	**215.00**	48.20	**22.20**	156.80	**0.91**	0.76	0.76	**0.91**	0.83	0.88	0.84	0.86	0.81
	**(15.49)**	(36.57)	**(16.45)**	(37.49)	**(0.07)**	(0.18)	(0.18)	**(0.07)**	(0.10)	(0.07)	(0.08)	(0.06)	(0.12)
3	214.20	47.00	23.00	158.00	0.90	0.77	0.77	0.90	0.83	0.88	0.84	0.86	0.81
	(12.34)	(32.44)	(13.87)	(33.14)	(0.06)	(0.16)	(0.16)	(0.06)	(0.09)	(0.06)	(0.07)	(0.06)	(0.11)
4	209.40	40.80	27.80	164.20	0.88	0.80	0.80	0.88	0.84	0.86	0.84	0.86	0.82
	(7.57)	(19.46)	(8.87)	(20.19)	(0.04)	(0.10)	(0.10)	(0.04)	(0.06)	(0.03)	(0.03)	(0.02)	(0.05)
5	214.00	**29.60**	23.20	**175.40**	0.90	**0.86**	**0.86**	0.90	**0.88**	**0.89**	**0.88**	**0.89**	**0.87**
	(10.05)	**(12.20)**	(10.73)	**(13.26)**	(0.04)	**(0.06)**	**(0.06)**	(0.04)	**(0.04)**	**(0.04)**	**(0.02)**	**(0.02)**	**(0.02)**
6	212.80	47.00	24.40	158.00	0.90	0.77	0.77	0.90	0.83	0.87	0.84	0.86	0.81
	(10.03)	(34.18)	(11.87)	(36.15)	(0.05)	(0.17)	(0.17)	(0.05)	(0.10)	(0.04)	(0.06)	(0.04)	(0.09)
7	205.20	45.20	23.20	168.60	0.90	0.78	0.78	0.90	0.83	0.88	0.85	0.86	0.82
	(16.89)	(29.35)	(9.73)	(41.88)	(0.04)	(0.15)	(0.15)	(0.04)	(0.08)	(0.04)	(0.06)	(0.04)	(0.10)
8	209.00	46.00	28.20	159.00	0.88	0.77	0.77	0.88	0.83	0.85	0.83	0.85	0.80
	(12.02)	(36.59)	(11.05)	(37.99)	(0.05)	(0.18)	(0.18)	(0.05)	(0.10)	(0.03)	(0.07)	(0.04)	(0.11)
9	205.20	39.60	25.40	172.00	0.89	0.81	0.81	0.89	0.85	0.87	0.85	0.86	0.84
	(17.20)	(25.68)	(10.55)	(34.89)	(0.05)	(0.13)	(0.13)	(0.05)	(0.07)	(0.03)	(0.05)	(0.04)	(0.07)

Mean and standard deviation (in parenthesis) obtained on five cross-validation test sets (see Table 2) are shown for each model. *Acc.* - Accuracy. Best average values are shown in bold font.

**Figure 5:**
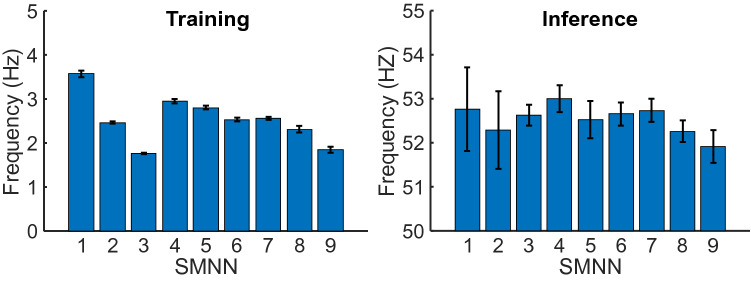
Running time comparison for neural network architectures SMNN 1–9. Mean training and inference frequency (number of samples per second) are shown for each model. Error bars denote standard deviation (SD).

**Figure 6:**
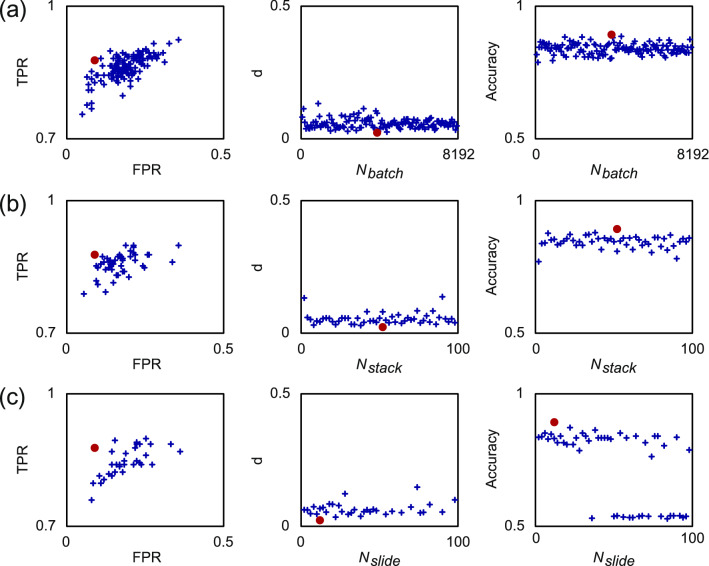
Results of hyperparameter tuning obtained with SMNN 5. (**a**) Batch size $N_{batch}$, (**b**) number of frames per input vector $N_{stack}$, and (**c**) offset between two consecutive input vectors $N_{slide}$. Left: TPR vs. FPR scores for hyperparameter tuning (the value closest to the TPR = 1 and FPR = 0 corresponds to the best perfromance). Middle: distance $d = \sqrt{\left( 1-\mathrm {TPR}\right) ^2 + \left( \mathrm {FPR}\right) ^2}$ with respect to TPR = 1 and FPR = 0 (the lowest value corresponds to the best performance). Right: classification accuracy (the highest value corresponds to the best performance). Red dot denotes the parameter with the best performance.

Runtime comparison of SMNN 1–9 architectures is presented in Fig. 5 where we show average training (left) and inference (right) frequency for each model. For this study, a CPU implementation (36 Core, 2.30 GHz) implemented with PyTorch is used.

The advantage of shallow networks is that the training time is short compared to deep learning architectures. For our proposed networks, the training frequency on average varies from $3.57 \pm 0.07$ to $1.76 \pm 0.02$ samples per second. Given that each training set contains about 900 training samples (see Table 2), this results to 4.2–8.5 min of training time.

Inference (prediction) runtime is one order of magnitude faster than training time and the inference frequency on average varies from $51.92 \pm 0.37$ to $53.0 \pm 0.31$ samples per second, which leads to $\approx 19$ *ms* of inference time per one sample (Note that this value holds only if the sample (input vector) is already copied into the RAM, otherwise the inference is about $44\ \mathrm{ms}$ per sequence.).

## Discussion

In this section, we discuss our results in the context of the state-of-the-art ML driven methods^28^. Our current study provides a simple, straight-forward pipeline for a computer-based GMA. The infant’s skeleton is used for providing features. This proves to be a major advantage to many other methods, where features are based upon wavelet functions, power-spectrum, or hand-crafted statistics, since the skeleton can be more easily interpreted by humans.

**Table 5:** Comparison of our approach to the state of the art methods from other studies.

Study	Classification	Acc. (%)	Sens. (%)	Spec. (%)
Current study	FM+ vs. FM−	88	88	88
Adde et al.^54^	FM+ vs. FM−		90	80
Machireddy et al.^55^	FM+ vs. FM−	70		
Tsuji et al.^39^	Normal (WMs, FMs) vs. Abnormal (CS, PR)	84.52		
Adde et al.^40^	CP vs. no-CP		85	88
Karch et al.^33^	CP vs. no-CP		90	96
Philippi et al.^56^	CP vs. no-CP		90	95
Orlandi et al.^29^	CP vs. no-CP	92.13		
Ihlen et al.^57^	CP vs. no-CP	87	92.7	81.6
Meinecke et al.^58^	Healthy vs. at-risk	73	100	70
Heinze et al.^59^	Healthy vs. pathologic	89.66		
Rahmati et al.^60^	Healthy vs. affected	87		
Rahmati et al.^61^	Healthy vs. affected	91		
Stahl et al.^62^	Impaired vs. unimpaired	93.7	85.3	95.5
Dai et al.^63^	Normal vs. abnormal	93.3	95	91.7
McCay et al.^64^	Normal vs. abnormal (synthetic data)	87.05		
Raghuram et al.^65^	Motor-impairment vs. no-motor-impairment	66	95	95
Gao et al.^66^	Typical development vs. abnormal movements	79		
Doroniewicz et al.^67^	Normal WM vs. PR movements	80.93		

The upper part of the table presents studies focusing on fidgety movements.

First, we discuss our results in light of the dataset. The intra-rater reliability were $\kappa = 85.4 \pm 0.1$% and $\kappa = 95.4 \pm 0.1$% respectively for the two assessors. A test–retest kappa of 0.85–0.95, rated on a series of merely 5-s clips (for which the assessors are not trained for), although not comparable to the actual intra-rater reliability of the respective GMA assessor, is strikingly high. To the best of our knowledge, it is the very first study demonstrating that well-trained and experienced GMA assessors are able to reliably classify the GMs by watching just 5 s of the infant’s natural movements, both at the inter-rater level (Cohen’s kappa $\kappa = 0.97$ between the two assessors), and, at the within-rater level. Nevertheless, it must be stressed, that standard GMA requires observation of an infant’s movements of at least 2–5 min^15^. In fact, a classification by an AI tool at the individual level, e.g. to evaluate whether an infant presents fidgety movements or not, must also be based on the accumulated ratings of the infant movement sequences over time, no matter how short a single judgment unit is chosen by the algorithm. No classification on the GMs, neither by human nor by computer, shall ever be drawn from a single 5-s video. Given the excellent inter- and intra-rater reliabilities, in the current study, we only included the snippets that were identically rated by both raters for machine learning, which shall maximize the reliability and validity of the dataset. As emphasized, from the clinical perspective, GMA is not about the 5-s behavior of an infant, but the overall movement pattern of an individual. For example, a typically developing infant at the “fidgety age” does not necessarily present FMs, nor the same intensity of the FMs, all the time. As shown in our dataset, a very small fraction of snippets from the typical fidgety age period (T5-7; 9 out of 946 snippets) were rated by both assessors unanimously as “FM−”, verifying a normal phenomenon that typically developing infants during the typical fidgety period do not demonstrate FMs at all times, although their predominant movement pattern is FM.

From a technological perspective, comparing results applying different methods proves to be difficult in general. Due to the confidentiality regulations protecting the participants, no common dataset yet exists for evaluating and collating performances of the different machine learning approaches. Recent attempts have been made with artificial data^64^, where artificial 3D models of infants are reconstructed based on recordings. However, even these authors themselves find performance differences in the original and artificial data. To compare and discuss this problem, we compiled a table of the state-of-the-art algorithms (Table 5).

Two studies used full pose recovery based on passive measurements^64,67^. McCay et al.^64^ used artificial data made up from “normal” and “abnormal” participants; As feature vector binned joint movements are used. Doroniewicz et al.^67^ analyzed 31 participants to distinguish normal and abnormal (i.e., poor repertoire) writhing movements. The feature vector holds information about the movement’s area, movement’s shape, and the center of the movement’s area. To the best of our knowledge, our study is the first that uses full pose recovery based on passive, single camera video streams with an easy to understand and analyzable feature vector that does not require further pre-processing.

In this work, we focused on the detection of fidgety movements. As mentioned before, since no common dataset is available, the results from the various studies analyzing heterogeneous samples are hardly comparable to each other (see Table 5). In some cases, sample characteristics are generally missing. For instance, in a handful of existent studies focusing on fidgety movements, despite their technical merits, Machireddy et al.^55^ and Tsuji et al.^39^ omitted certain essential information about all, or a part of, the participants (e.g., gestational age, medical condition), raising question on the validity of such studies concerning the fundamental concepts of GMA. Adde et al.^54^ provided detailed information about their participants. As they analyzed movements from a convenience clinical sample, including preterm and term infants (i.e., pooling both the normal and abnormal GM patterns), their dataset is radically different from the one used in the current study—the normal age-specific movement patterns acquired from a group of prospectively sampled typically developing infants.

## Conclusion

This study proposes a novel machine learning algorithm to detect an age-specific movement pattern, the fidgety movements, in a prospective sample of typically developing infants. Participants were recorded using a passive, single camera RGB video stream. No further sensors were needed. According to the GMA procedure, the dataset was annotated by two well-trained and experienced GMA assessors. The inter- and intra-rater reliability between the assessors were excellent. Using OpenPose^45^, with the validated dataset, the infant full pose was recovered from the video stream in form of a 25-point skeleton. This skeleton was used as input vector for shallow multilayer neural network (SMNN) architectures. No further pre-processing was needed. The input vector was well accessible to humans. An ablation study was performed to justify proposed network’s architecture and its hyperparameters. We show, for the very first time, that the SMNN is sufficient to discriminate fidgety movements from non-fidgety movements in a validated sample of age-specific typical movements with an average classification accuracy of $88 \%$. Another advantage of the proposed network architectures is relatively short training (4–9 min for about 900 training samples) and inference time ($\approx$ 19 ms per sample).

To circumvent the shortage of a large dataset, which can pose a problem, we may investigate in the future the feasibility of using home-recordings to serve the automated GMA. The non-standard home videos will result in heterogeneous datasets (e.g., different backgrounds, variable distances, and perspectives to the infant) that is particular challenging for computer vision and machine learning approaches. As pointed out by other scientists, neither human nor computer rating could ever reach an unrealizable one-hundred-percent accuracy^68^. At the time, there is no question of replacing human clinical reasoning, but rather how to augment technological approaches to assist and strengthen classic GMA^69^. This is particularly relevant to resource limited settings where clinics are very busy and study personnel tend to be strained; computer-based approaches may alleviate the work load ensuing fatigue and affecting study staff, thus enhancing performance and overall quality of the GMA. The technology will also facilitate interpretation of large datasets. In summary, computer-based solutions will complement classic GMA to consistently perform accurate and efficient screening and diagnosis that may become universally accessible in daily clinical practice in the future.

## Appendix

See Fig. 6.
